# Case Report: Pulmonary fibroleiomyomatous hamartoma presenting as a giant exophytic multilocular cystic lesion

**DOI:** 10.3389/fmed.2026.1924103

**Published:** 2026-08-10

**Authors:** Fei Han, Zongming Wang, Xianchao Zhang, Xin Wang

**Affiliations:** 1 School of Clinical Medicine, Shandong Second Medical University, Weifang, Shandong, China; 2 Department of Thoracic Surgery, Central Hospital Affiliated to Shandong First Medical University, Jinan, Shandong, China; 3 Department of Pathology, Central Hospital Affiliated to Shandong First Medical University, Jinan, Shandong, China; 4 Department of Respiratory and Critical Care Medicine, Central Hospital Affiliated to Shandong First Medical University, Jinan, Shandong, China

**Keywords:** cystic lung lesion, immunohistochemistry, lung, pulmonary fibroleiomyomatous hamartoma, rare disease

## Abstract

Pulmonary fibroleiomyomatous hamartoma (PLH) is a rare benign subtype of pulmonary hamartoma. It typically presents on imaging as a well-circumscribed solid pulmonary nodule. The lesion can arise in any region of the lung. There is a slight female predominance, and most patients are asymptomatic or present with mild, non-specific respiratory symptoms such as cough and sputum production. In most cases, the lesion is incidentally detected during routine health examinations or imaging studies performed for unrelated conditions. This case report describes a 55-year-old woman in whom a cystic abnormal lesion with areas of air lucency was incidentally detected in the right upper lobe during a routine health examination. The lesion appeared as a large space-occupying mass with cystic wall enhancement on imaging, and a congenital cystic lesion was initially suspected. After admission, the patient underwent thoracoscopic wedge resection of the right upper lobe lesion. Intraoperatively, the tumor demonstrated a pedunculated exophytic growth pattern with a narrow stalk. Postoperative histopathological examination revealed a biphasic architecture composed of epithelial and mesenchymal components. Immunohistochemical findings, together with the histopathological features, favored the diagnosis of pulmonary fibroleiomyomatous hamartoma. The patient recovered uneventfully after surgery, and no evidence of recurrence was observed during the available follow-up period. To our knowledge, this is the first reported case describing a pulmonary lesion favoring the diagnosis of pulmonary fibroleiomyomatous hamartoma that presents predominantly as a giant exophytic multilocular cystic lesion. The patient had a previous history of hysterectomy for uterine leiomyoma, and the lesion showed positive expression of estrogen receptor (ER) and progesterone receptor (PR), suggesting a potential hormonal association. This case highlights that pulmonary fibroleiomyomatous hamartoma should be considered in the differential diagnosis of pulmonary lesions in female patients with a history of uterine leiomyoma, even when imaging findings demonstrate atypical cystic features, to reduce the risk of misdiagnosis.

## 1 Introduction

Pulmonary fibroleiomyomatous hamartoma (PLH) is a rare benign pulmonary tumor. It was first reported by Logan et al. (1). PLH was initially considered to occur exclusively in women of reproductive age and was frequently associated with uterine leiomyoma; however, subsequent reports have also documented cases in male patients (2). Clinically, PLH is usually asymptomatic or presents with mild, non-specific respiratory symptoms, and it is most often incidentally detected during routine health examinations. Laboratory tumor markers are typically negative. Radiologically, PLH commonly presents as solitary or multiple well-circumscribed pulmonary nodules (3). In contrast, the present case demonstrated a predominantly cystic appearance on imaging, along with a completely exophytic pedunculated growth pattern. The unique exophytic multilocular cystic phenotype observed in this case may broaden the recognized imaging spectrum of pulmonary fibroleiomyomatous hamartoma and provide additional considerations for the differential diagnosis of atypical pulmonary lesions.

## 2 Case presentation

### 2.1 Patient information

**Figure 1:**
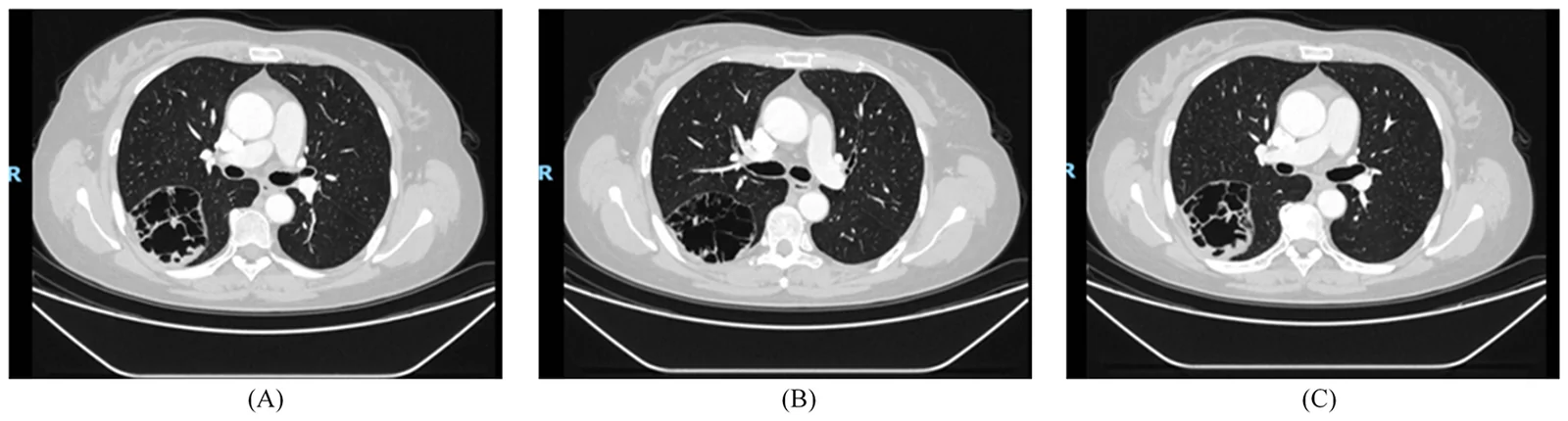
Chest computed tomography (CT) findings of the pulmonary lesion. **(A–C)** CT images revealed a round-to-oval abnormal density lesion in the right upper lobe, containing multiple cystic spaces with internal septations. The lesion measured approximately 69 mm × 64 mm in maximum cross-sectional dimensions. The lesion was relatively well circumscribed, with thickened septa and cyst walls showing irregular thickening and relatively homogeneous moderate enhancement. No surrounding exudation or satellite lesions were observed.

A 55-year-old woman with a history of hysterectomy for uterine leiomyoma at the age of 51 was admitted to our hospital after a pulmonary lesion was detected during routine physical examination. She was regarded as postmenopausal at the time of diagnosis. She was asymptomatic and denied cough, sputum production, hemoptysis, chest pain, dyspnea, fever, chills, weight loss, fatigue, night sweats, or other constitutional symptoms. Chest computed tomography (CT) revealed a well-circumscribed, round abnormal-density lesion in the right upper lobe measuring approximately 69 mm × 64 mm. The lesion contained multiple cystic spaces separated by internal septa. The cyst walls and septa were thickened, with focal irregular wall thickening and moderate homogeneous enhancement on contrast-enhanced imaging. Additionally, an oval air-containing component measuring approximately 11 mm in maximal diameter was observed within the lesion, with a well-defined margin and no significant enhancement (Figure 1). Based on the radiological findings, a developmental cystic lesion was initially considered. The patient's medical history was notable for a previous hysterectomy for uterine leiomyoma. She had no history of hypertension, diabetes mellitus, coronary artery disease, asthma, tuberculosis, hepatitis, or other major chronic illnesses. She was a lifelong non-smoker and denied alcohol consumption.

On admission, physical examination was unremarkable. Vital signs were within normal limits, and no abnormalities were detected on cardiopulmonary, abdominal, or neurological examination. Laboratory investigations, including routine blood tests and serum biochemical analyses, were within normal ranges.

### 2.2 Surgical findings and pathological examination

Given the indeterminate nature of the lesion and the inability to exclude malignancy preoperatively, the patient underwent video-assisted thoracoscopic surgery (VATS) with wedge resection of the right upper lobe lesion.

Intraoperatively, a cystic-solid mass measuring approximately 7 cm in diameter was identified external to the posterior segment of the right upper lobe. The lesion exhibited a pedunculated exophytic growth pattern and was connected to the lung parenchyma by a narrow stalk arising from the posterior segment of the right upper lobe. The stalk was transected and closed using an endoscopic stapling device, and the mass was completely excised. Intraoperative frozen-section examination suggested a benign smooth muscle-derived neoplasm.

#### 2.2.1 Gross pathological findings

**Figure 2:**
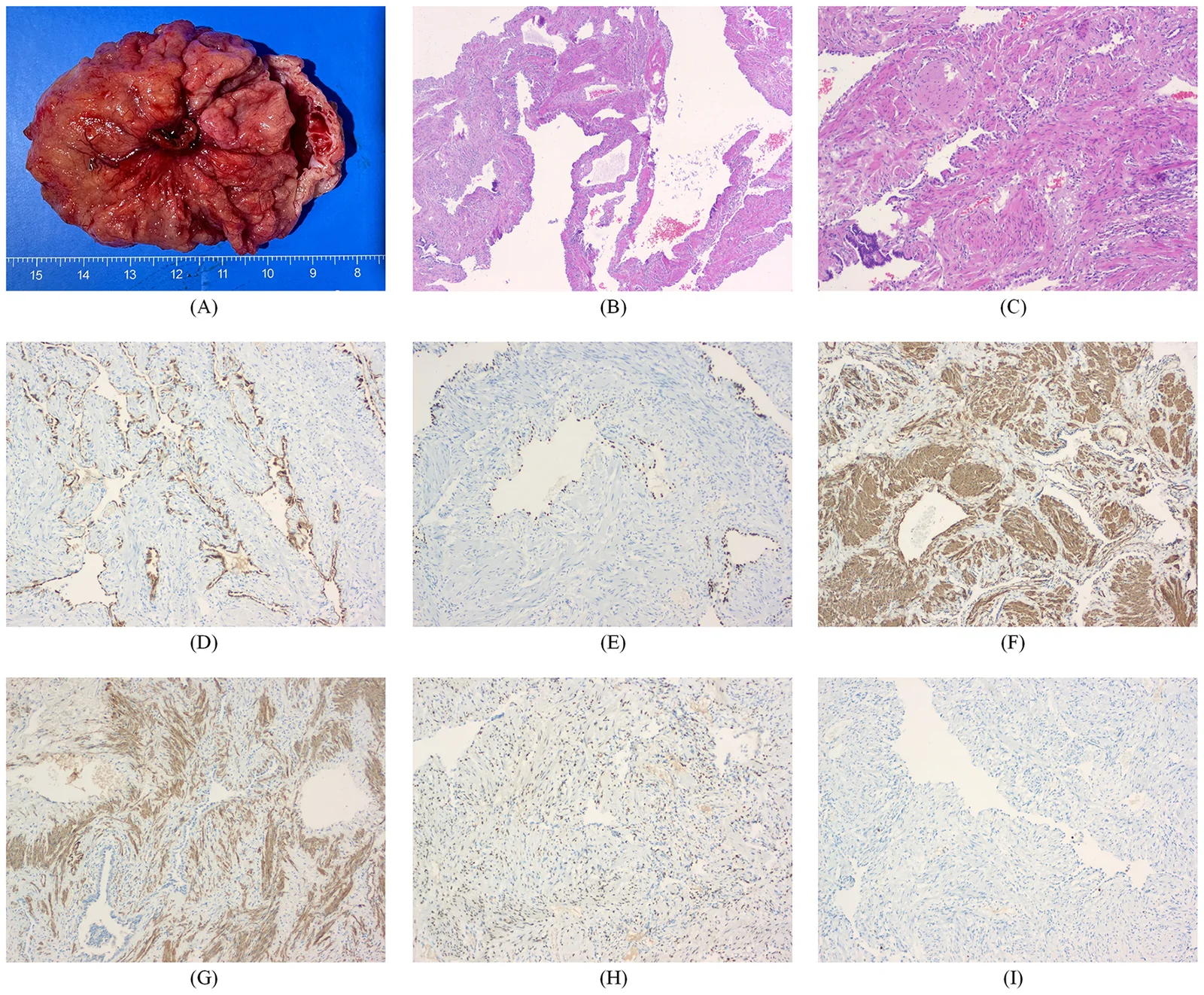
Histopathological and immunohistochemical findings. **(A)** Gross examination of the resected specimen revealed a large cystic mass. **(B)** Low-power photomicrograph demonstrating a cystic and solid lesion [hematoxylin and eosin (H&E), × 40]. **(C)** Intermediate-power photomicrograph showing a biphasic lesion composed of epithelial and mesenchymal components. The epithelial component is lined by a single layer of flattened to cuboidal epithelial cells, whereas the mesenchymal component consists predominantly of well-differentiated smooth muscle cells admixed with fibrous stroma (H&E, × 100). **(D)** The epithelial component shows diffuse immunoreactivity for pan-cytokeratin (pan-CK) (IHC, × 100). **(E)** The epithelial cells demonstrate positive nuclear staining for thyroid transcription factor-1 (TTF-1) (IHC, × 100). **(F)** The spindle cell component showed partial positivity for H-caldesmon (IHC, × 100). **(G)** The spindle cells show partial immunoreactivity for smooth muscle actin (SMA) (IHC, × 100). **(H)** The spindle cells show nuclear immunoreactivity for estrogen receptor (ER) (IHC, × 100). **(I)** Ki-67 immunostaining reveals a low proliferative index in both the epithelial and spindle cell components (IHC, × 100).

Gross examination revealed a solitary cystic nodular mass measuring 7.0 cm × 5.0 cm × 1.5 cm. The external surface was smooth, and a pedicle measuring approximately 1.0 cm in length and 0.8 cm in diameter was present. On sectioning, the lesion showed a multilocular cystic architecture with cyst wall thickness ranging from 0.1 to 1.0 cm. Most of the cystic lining was smooth; however, focal papillary projections were identified and were scattered throughout the lesion (Figure 2).

#### 2.2.2 Histopathological examination

Microscopically, the tumor exhibited a biphasic architecture composed of epithelial and mesenchymal components. The epithelial component consisted of a single layer of flattened to cuboidal cells. The mesenchymal component was predominantly composed of well-differentiated smooth muscle cells and fibrous connective tissue arranged in fascicular, interlacing, and whorled patterns, with scattered adipose tissue. Focally, the stromal component formed coarse papillary structures covered by epithelial cells, resembling adenofibromatous architecture. Mild scattered inflammatory cell infiltration was observed within the stroma.

#### 2.2.3 Immunohistochemical analysis

For the smooth muscle component: SMA (partially positive), Desmin (partially positive), H-caldesmon (partially positive), ER (positive), and PR (positive). For the epithelial component: TTF-1 (positive) and cytokeratin (CK, positive). Other markers: CD34 (positive in vascular endothelium), Bcl-2 (weakly positive), CD68 (scattered positivity), Ki-67 (approximately 1%), and p53 (less than 1% positive). CR, ALK, STAT6, HMB45, Melan-A, S-100, and D2-40 were all negative (Figure 2).

### 2.3 Diagnosis and outcome

Based on the histopathological features and immunohistochemical profile, the diagnosis of Pulmonary fibroleiomyomatous hamartoma was favored. The patient recovered uneventfully without any discomfort. During regular follow-up, no evidence of tumor recurrence was observed.

### 2.4 Timeline

Key clinical events are summarized in Table 1.

**Table 1:** Timeline of key clinical events.

Timeline	Event
One day prior to admission	A routine physical examination revealed a cystic lesion with abnormal attenuation in the right upper lobe of the lung
Day of admission	Chest computed tomography (CT) revealed a well-circumscribed multiloculated cystic lesion (69 mm × 64 mm) in the right upper lobe with thickened septa and cyst wall showing moderate homogeneous contrast enhancement. The patient was admitted for further evaluation and treatment
Hospital day 1	Routine laboratory investigations, including complete blood count, serum biochemistry, and coagulation profile, were all within normal limits
Hospital day 2	Video-assisted thoracoscopic wedge resection of the right upper lobe was performed. Intraoperatively, a pedunculated cystic lesion measuring approximately 7 cm was identified arising from the posterior segment of the right upper lobe and was completely resected using an endoscopic stapling device. Frozen-section examination suggested a benign smooth muscle tumor
Postoperative day 4	The postoperative course was uneventful, and the patient was discharged
Postoperative day 7	Histopathological examination and immunohistochemical staining revealed findings favoring the diagnosis of pulmonary fibroleiomyomatous hamartoma
Three months post-surgery	Follow-up chest CT showed no evidence of recurrence

## 3 Discussion

Pulmonary fibroleiomyomatous hamartoma (PLH) is a rare benign pulmonary tumor. A literature review suggests that fewer than 30 cases have been reported in China and internationally, most of which are presented as case reports. The hallmark pathological feature is the excessive proliferation of well-differentiated smooth muscle cells within the lung, exhibiting a tumor-like growth pattern, accompanied by interspersed mature bronchial or alveolar epithelial components.

Accordingly, radiologically, PLH typically presents as nodular lesions of varying sizes with smooth margins (4, 5). On contrast-enhanced CT, mild to moderate enhancement may be observed. Fat, cartilage, and calcification are generally absent within the lesions, which may lead to frequent misdiagnosis as metastatic tumors (6, 7). Histologically, the lesions appear as firm, gray-white, whorled nodules composed of interlacing bundles of mature smooth muscle cells and collagen fibers, often associated with slit-like or glandular structures formed by invaginated respiratory epithelium.

However, the present case significantly expands the classical understanding of PLH. Its unique features can be summarized in three aspects. First, radiologically, the lesion manifested as a giant multilocular cystic mass measuring 69 mm × 64 mm, with internal septations and irregular thickening and moderate enhancement of the cyst wall, rather than the typical solid nodule. This may reflect secondary cystic dilatation caused by mucinous or myxoid degeneration within fibromuscular bundles and epithelial-lined clefts. Second, the tumor demonstrated a completely pedunculated exophytic growth pattern, measuring approximately 7 cm, connected to the lung parenchyma by a narrow stalk of 1 cm in length and 0.8 cm in diameter, which is distinctly different from the usual intrapulmonary infiltrative or expansile growth patterns of hamartomatous lesions. Third, histologically, focal stromal papillary structures lined by a single layer of epithelium were observed, resembling fibroadenoma-like morphology. These three atypical features collectively posed significant diagnostic challenges on preoperative imaging and constitute the major academic novelty and clinical value of this case report.

In terms of differential diagnosis, Benign metastasizing leiomyoma (BML) was an important consideration in the present case due to the patient's previous history of hysterectomy for uterine leiomyoma. BML typically occurs in women with a history of uterine leiomyoma resection, with bilateral multiple well-circumscribed pulmonary nodules being the most common radiological presentation (8). Histologically, BML is characterized by a proliferation of bland, mature smooth muscle cells without true epithelial lining structures. The smooth muscle cells typically express smooth muscle markers on immunohistochemical staining, and estrogen receptor (ER) and progesterone receptor (PR) expression is frequently observed. Unlike the present case, BML generally lacks the prominent biphasic epithelial–mesenchymal architecture composed of epithelial and mesenchymal components. However, the relationship between PLH and BML remains controversial. Previous studies have suggested that these entities may share overlapping biological characteristics, including smooth muscle differentiation and hormone receptor expression, and may represent different phenotypic manifestations within a spectrum of pulmonary leiomyomatous proliferations (9). Further molecular pathological investigations, including clonality analysis and genetic profiling, may help clarify the biological relationship between these two entities.

In contrast, the present lesion demonstrated a distinctive biphasic composition consisting of mature smooth muscle proliferation and epithelial components, with expression of epithelial markers (CK and TTF-1), which are not characteristic of typical BML. Therefore, the overall clinicopathological findings in this case favored the diagnosis of PLH.

The present case underwent whole-exome sequencing of the tumor tissue, which identified a missense variant in the TSC2 gene (c.2365G>A, p.Val789Ile). This variant is extremely rare in population databases and is currently classified in ClinVar as a variant of uncertain significance (VUS). Because no matched peripheral blood or normal tissue was available for germline validation, and no analysis of a “second-hit” event was performed in the tumor tissue, a causal relationship between this variant and the lesion cannot be established. Therefore, this variant should be interpreted cautiously and may represent a background genetic alteration with currently unclear biological significance. In addition, the patient showed no clinical manifestations of Tuberous Sclerosis Complex (TSC) (10, 11). Immunohistochemical staining for melanocytic markers, including HMB45 and Melan-A, was negative, which is not consistent with the immunophenotype of TSC-associated Lymphangioleiomyomatosis (LAM) or PEComa.

Overall, the morphological and immunohistochemical features of this lesion favor the diagnosis of pulmonary fibroleiomyomatous hamartoma. However, the predominantly giant cystic imaging presentation and the pedunculated exophytic growth pattern suggest a rare and incompletely recognized variant. This case posed significant preoperative diagnostic challenges. It highlights that in adult patients with solitary giant cystic lung lesions—particularly those that are well-circumscribed, slowly growing, and lack invasive features—PLH should be considered even when imaging findings are atypical. Given its benign biological behavior, complete surgical excision is associated with an excellent prognosis, and recurrence is exceedingly rare. At present, surgical resection remains the most effective treatment for PLH (2). In this case, complete removal was achieved via thoracoscopic wedge resection, and the patient recovered well without recurrence during follow-up.

The main limitation of this case report is the relatively short follow-up duration. Although no recurrence or disease progression was observed during the current follow-up period, longer-term follow-up is necessary to further elucidate the clinical behavior and prognosis of PLH. Therefore, continued follow-up will be planned to monitor for potential recurrence.

## 4 Conclusion

In summary, this case highlights an unusual phenotypic presentation of a pulmonary lesion favoring the diagnosis of pulmonary fibroleiomyomatous hamartoma. The present lesion is clinically rare, with atypical radiological manifestations that may lead to significant diagnostic difficulty. For clinicians, PLH should be included in the differential diagnosis when encountering atypical cystic pulmonary lesions, particularly in patients with a history of uterine leiomyoma, in order to improve diagnostic accuracy and support appropriate clinical decision-making. Furthermore, this case may serve as a useful reference for clinicians and pathologists to enhance their understanding of this rare entity.

## Data Availability

The original contributions presented in this study are included in the article/supplementary material. Further inquiries can be directed to the corresponding author.
